# Early posthatch feeding influences small intestine development in broiler chickens

**DOI:** 10.1016/j.psj.2025.105861

**Published:** 2025-09-18

**Authors:** Andrzej Gaweł, Kamila Bobrek, Jan P. Madej

**Affiliations:** a Department of Epizootiology and Clinic of Birds and Exotic Animals, Wrocław University of Environmental and Life Sciences 50-366 Wrocław, Poland; b Department of Immunology, Pathophysiology and Veterinary Preventive Medicine, Wrocław University of Environmental and Life Sciences 50-375 Wrocław, Poland

**Keywords:** Gut, Hatching window, Early feeding, Broiler, Poultry

## Abstract

The height of the intestinal villi and the consequent small intestinal surface area are factors that limit broiler growth rate after hatching. Previous studies indicated that in poultry, the greatest increase in relative small intestinal weights and villous heights occurs during the first week after hatching, with villous height continuing to increase in the following weeks of life. This study aimed to evaluate the impact of providing access to feed and water during the hatching window on small intestine development, structure, and morphometry. The experiment was conducted using 100,000 Ross 308 broiler eggs incubated under typical commercial hatchery conditions. Eggs in the experimental group were hatched in HatchCare hatchers (HC) with immediate access to feed and water, while those in the control group were hatched under standard conditions (ST). Samples of the duodenum, jejunum, and ileum were evaluated from each group on days 1, 7, 21, and 35 after hatching. Villous height, width, and surface area (VSA), as well as crypt depth, villus-to-crypt ratio (VCR), and goblet cell number, were morphometrically assessed. Early access to feed increased significantly the VSA in the duodenum and ileum of the HC group on day 7. It also initially increased crypt depth in the jejunum and ileum during the first week of age, followed by a decrease in the jejunum on days 21 and 35. Early feeding had no influence on VCR from day 7 onward in all intestinal segments. The number of goblet cells did not differ in the duodenum and jejunum, but in the ileum, the HC group had fewer goblet cells than the ST group on days 21 and 35. It can be concluded that early feeding has the most beneficial effects on small intestine development during the first week after hatching. However, these effects did not persist until the end of broiler life cycle.

## Introduction

The optimal condition of the intestine is crucial to growth and weight gain in poultry. Previous studies on gastrointestinal tract development posthatch have indicated that in poultry, the greatest increase in relative small intestinal weights and villous heights occurs during the first week after hatching (Jha et al., 2019; Wijtten et al., 2012). Following this, the relative weight of the small intestine gradually decreases, while villous height continues to increase (Wijtten et al., 2012). This process supports steady growth of the intestinal absorptive capacity with a lower intestinal weight, which is vital for flying birds and is evolutionarily conserved in broilers (Wijtten et al., 2012). It can be concluded that chickens prioritize small intestine development during the first week of life. Therefore, early feed deprivation can lead to a decreased villus surface area (**VSA**) and shorter intestinal enterocytes, which in turn impairs nutrient utilization and growth (Noy et al., 2001). In contrast, early feeding stimulates gut development, enhances intestinal motility, and increases the passage of yolk sac content to the gut for subsequent absorption (Noy et al., 2001).

Under large-scale farm conditions, chicks hatch over a 24–48 h period, known as the hatching window. Chicks that hatch first face an unfavorable situation, as they experience a prolonged fasting period and potential dehydration, which is worsened by suboptimal temperature conditions (Noy and Uni, 2010). Various treatments within the hatchery and subsequent transport to broiler farms often result in a delay of up to 72 h before the chicks receive their first feed and water (Willemsen et al., 2010). During this time, hatchlings lose weight at a rate of approximately 4 g per 24 h due to moisture loss as well as utilization of yolk and pectoral muscle (Careghi et al., 2005; Halevy et al., 2003; Tona et al., 2003; van de Ven et al., 2009). In addition, delayed access to feed and water increases susceptibility to pathogens (Dibner et al., 1996), reduces immune performance (Juul-Madsen et al., 2004), delays muscle and satellite cell development (Gaweł et al., 2022; Halevy et al., 2000; Moore et al., 2005), and impairs maturation and colonization of the bursa of Fabricius and gut-associated lymphoid tissue (GALT) in the hindgut (Shira et al., 2005).

Chickens are precocial animals; therefore, they naturally begin feeding immediately after hatching (Henderson et al., 2008; Noy and Uni, 2010). Early posthatch feeding in hatching chambers (referred to in this paper as HatchCare technology) provides access to feed and water as soon as possible, enabling optimal utilization of the chickens’ genetic potential from the first hours after hatching. To date, this strategy has been shown to enhance growth rate and increase the proportion of breast muscle at the end of the fattening period (Gaweł et al., 2022; Halevy et al., 2000; Hassan et al., 2023; Noy and Sklan, 1999), stimulate gut development (Ao et al., 2012; Lamot et al., 2016; Li et al., 2022; Roto et al., 2016; Wang et al., 2020), stimulates thermoregulatory ability in long term (Wijnen et al. 2022), and intensify metabolism by triggering digestive enzyme secretion and increasing absorption of glucose, methionine, and oleic acid by the small intestine (Halevy et al., 2000; Sklan and Noy, 2000; Taha-Abdelaziz et al., 2018). However, there are also reports indicating that early feeding has no effect on body weight or on the weight of selected organs on day 35 after hatching (Abbas Yaseen Hussein and Jawad 2023).

This study aimed to evaluate the impact of providing access to feed and water during the hatching window on the development, structure, and morphometry of the small intestine and on the yolk sac weight. Data from the same experiment regarding the body weight of birds (**BW**), weight of the breast muscles and diameter of breast-muscle fibers were published by Gaweł et al. (2022). It was found that early feeding significantly improves the BW on day 7 after hatching but the final BW on day 35 was only slightly (5 %) higher in the HC group compared to the control group. The temporary increase of the breast-muscle fibers (D7 and D21) was also found, but no improvement in breast muscle weight was achieved in broilers at the end of the fattening period (on D35) (Gaweł et al. 2022). Considering the improvement in the above-mentioned production parameters at selected time points, we checked whether they could result from an enhancement of the process of nutrient absorption from the intestine due to morphological changes occurring in this organ.

## Materials and methods

### Experimental design

Ross 308 broiler breeder eggs with an average weight of 64 g (range 60–67 g) derived from one 42-wk-old breeder flock and were incubated under large-scale commercial hatchery conditions (ModernHatch, Niemodlin, Poland) using HatchTech incubators. Fifty thousand eggs were placed in typical hatcher chambers (HatchTech) as the control named standard group (**ST**), while 50,000 eggs were placed in HatchCare hatchers, which provide immediate access to feed and water in an environment with fresh air and LED illumination (Incubation Technology that Delivers Consistently Superior Chick Quality) as the experimental group (**HC**). The hatchability in the HC group was 89.9 % and did not differ significantly from the ST (88.5 %) group. The chicks were removed from the hatcher chambers 21 days and 12 h after the start of incubation, resulting in a hatching window lasting 48–55 h, and then transferred to poultry houses on commercial farm. The birds were reared under identical environmental conditions and fed commercial diets (starter, grower, and finisher) following animal welfare recommendations outlined in the European Union Directive 86/609/EEC. Appropriate husbandry conditions were maintained, including continuous monitoring of stocking density, litter quality, ventilation, and other factors (Sobolewska et al., 2017, Aviagen 2019) Throughout the entire 35-day experiment, the birds had constant access to feed and water. The study was conducted with the approval of the Local Ethics Committee for Animal Experiments (permission number 104/2017, Wrocław, Poland) and met the guidelines approved by the Institutional Animal Care and Use Committee (IACUC).

### Materials

Thirty chickens from the ST and HC groups were randomly selected on days 1 (**D1**), 7 (**D7**), 21 (**D21**), and 35 (**D35**) after hatching and subjected to necropsy. After sacrifice, the small intestine was removed, and the duodenum, jejunum, and ileum were dissected, measured, and weighed. Due to difficulties in completely removing intestinal contents on D1, the weight results obtained at this time point were considered unreliable and were excluded from further analysis. On day 1 the yolk sacs were removed and weighed and the result was correlated with the body weight of the bird. The yolk sac index was calculated according to the formula: yolk sac weight(g) / body weight(g) × 100 %.

For histological studies, samples of the small intestine—duodenum, jejunum, and ileum—were collected from eight birds in each group. Specimens were taken from the midpoint of the duodenum, the midpoint of the jejunum between the bile duct entry point and Meckel’s diverticulum, and the midpoint of the ileum between Meckel’s diverticulum and the ileocecal junction. The intestinal fragments were flushed with 0.9 % saline, fixed in 4 % buffered formaldehyde, and routinely embedded in paraffin. Sections (5 µm thick) of each tissue were stained with hematoxylin and eosin (H&E), using hematoxylin according to Mayer (Roth GmbH, Karlsruhe, Germany) and eosin (Poch S.A., Gliwice, Poland).

### Histomorphometry

Measurements were performed three times for each structure per bird and included the height and width of intestinal villi, intestinal crypt depth, and goblet cell number. The sections were examined and photographed under a Nikon Eclipse 80i light microscope (Nikon, Melville, NY, USA) equipped with a video camera and the NIS-Elements AR 2.30 software (Nikon). Villus height was estimated in cross sections of the intestine. Only intact villi that were perpendicularly sectioned through the midline axis were selected for measurement. Similarly the width was measured at half of their length. Based on these data, the average VSA was calculated using the formula provided by Sakamoto et al. (2000): VSA = (2π) × (VW/2) × (VH), where VW = villus width, and VH = villus height.

The depth of the intestinal crypts was defined as the invagination depth between neighboring intestinal villi and was measured from the base upward to the transition region between the crypt and the villus (Uni et al., 1998). The number (density) of goblet cells was calculated per 1 linear millimeter of epithelium covering the intestinal villi.

### Statistical analysis

The data collected during morphometric measurements were analyzed using Statistica 13.3 software (StatSoft Polska Sp. z o.o., Cracow, Poland). The significance of differences was assessed using the *t*-test for data conforming to a normal distribution, or the Mann–Whitney U test for data not conforming to a normal distribution. A value of *P* < 0.05 was considered significant and marked on graphs with a single asterisk, while *P* < 0.01 was marked with a double asterisk. The villus-to-crypt ratio (**VCR**) was calculated for each individual as the ratio of mean villus height to mean crypt depth, based on three measurements. Only these individual results were used to calculate the group mean.

## Results

### Weight and length

The average weights of the individual small intestine regions are shown in Table 1. Significantly higher values were observed in the jejunum on day 7 and in the ileum on day 35 in the HC group compared to the ST group.

**Table 1:** The weight (mean ± SD, n = 30) of the duodenum, jejunum, and ileum in the standard (ST) and early fed – HatchCare (HC) groups on days 7, 21, and 35 after hatching. Significant difference compared to ST (* *P* < 0.05; ** *P* < 0.01).

	Duodenum		Jejunum		Ileum	
	ST	HC	ST	HC	ST	HC
D7	3.29 ± 0.97	3.25 ± 1.12	3.89 ± 0.99	4.32 ± 1.33*	3.26 ± 1.25	3.64 ± 1.44
D21	9.92 ± 2.53	10.00 ± 2.90	17.12 ± 7.93	15.87 ± 6.10	13.89 ± 7.81	12.48 ± 5.29
D35	15.62 ± 4.82	16.22 ± 5.31	25.26 ± 5.49	27.81 ± 11.69	20.23 ± 5.04	22.49 ± 7.30*

The mean lengths of the small intestine regions are presented in Table 2. On day 1, the ileum in the HC group was significantly longer than in the ST group. However, on day 21, the length of the ileum, and on day 35, the length of the duodenum in the HC group were significantly shorter than those in the ST group.

**Table 2:** The length (mean ± SD, n = 30) of the duodenum, jejunum, and ileum in the standard (ST) and early fed – HatchCare (HC) groups on days 1, 7, 21, and 35 after hatching. Significant difference compared to ST (* *P* < 0.05; ** *P* < 0.01).

	Duodenum		Jejunum		Ileum	
	ST	HC	ST	HC	ST	HC
D1	8.86 ± 1.36	9.30 ± 1.52	20.77 ± 3.22	21.81 ± 4.63	19.10 ± 3.13	20.76 ± 4.14**
D7	18.65 ± 2.36	18.45 ± 3.06	44.82 ± 4.42	46.08 ± 4.67	44.97 ± 7.04	45.90 ± 4.55
D21	26.52 ± 3.70	26.45 ± 2.97	72.18 ± 8.66	70.80 ± 9.52	73.21 ± 9.56	68.48 ± 9.86**
D35	32.92 ± 3.83	31.51 ± 4.49*	84.39 ± 8.75	85.22 ± 10.31	83.54 ± 10.90	85.35 ± 10.29

The average weights of the yolk sacs measured on day 1, did not differ significantly between the HC (3.14 ± 1.28) and the ST (3.33 ± 1.54) group (data not shown). The yolk sac index in the HC group was 6.94 ± 2.86 and did not differ from that in the ST group (7.00 ± 2.71) (data not shown).

### Histology

During histological examination of the small intestines, no pathological changes were detected in either group at any of the time points studied.

### Histomorphometry

**Fig. 1:**
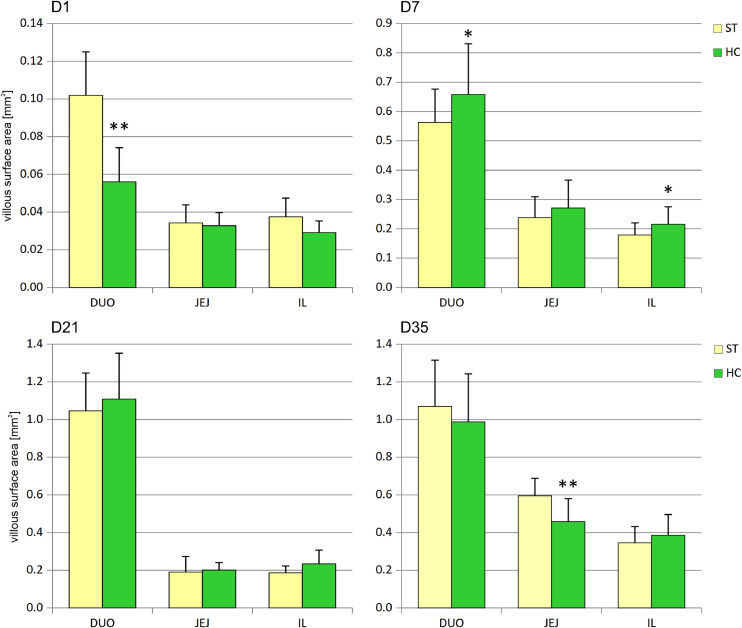
Effect of early feeding on average villus surface areas [mm^2^] in duodenum (DUO), jejunum (JEJ), and ileum (IL) (mean ± SD, n = 8). ST – standard, HC – HatchCare (early fed) group, D1 – day 1, D7 – day 7, D21 – day 21, and D35 – day 35 after hatching. Significant difference between groups (* *P* < 0.05; ** *P* < 0.01).

In the duodenum on day 1, a decrease in average VSA was observed in the HC group (Fig. 1), resulting from villus shortening and narrowing (Table 3). On day 7, VSA in the duodenum and ileum of the HC group increased significantly. On day 21, villus width increased in the jejunum and ileum of the HC group, but VSA did not change. On day 35, VSA in the jejunum of the HC group decreased, mainly due to villus shortening.

**Table 3:** Effect of early feeding on intestinal villi height and width on days 1, 7, 21 and 35 after hatching (mean ± SD, n = 8). HC – HatchCare (early fed) group, ST – standard group. Significant difference compared to ST (* *P* < 0.05; ** *P* < 0.01).

	Villus height (µm)		Villus width (µm)	
	ST	HC	ST	HC
D1				
Duodenum	381.5 ± 51.4	247.6 ± 72.4**	85.6 ± 17.4	72.1 ± 12.5**
Jejunum	226.9 ± 47.4	155.9 ± 22.3**	48.0 ± 8.6	67.4 ± 13.2**
Ileum	204.3 ± 35.6	158.1 ± 23.1**	58.2 ± 9.7	58.5 ± 8.0
D7				
Duodenum	1062.6 ± 123.5	1194.0 ± 167.8**	168.4 ± 24.1	175.1 ± 37.1
Jejunum	564.3 ± 131.8	513.6 ± 113.0	135.0 ± 27.4	164.8 ± 31.4**
Ileum	417.2 ± 47.8	464.8 ± 73.4*	135.9 ± 24.8	147.8 ± 30.9
D21				
Duodenum	1686.0 ± 141.4	1749.2 ± 149.9	196.9 ± 30.2	200.6 ± 36.6
Jejunum	529.9 ± 150.9	485.5 ± 69.7	111.9 ± 26.0	131.9 ± 17.3**
Ileum	510.7 ± 82.6	539.4 ± 104.8	116.5 ± 16.6	136.9 ± 29.3*
D35				
Duodenum	1578.5 ± 227.6	1480.6 ± 222.9	216.5 ± 42.3	211.9 ± 42.6
Jejunum	1054.4 ± 108.0	884.2 ± 143.7**	179.8 ± 23.6	164.3 ± 30.8
Ileum	695.2 ± 77.7	736.1 ± 131.1	158.3 ± 35.1	166.5 ± 32.8

**Fig. 2:**
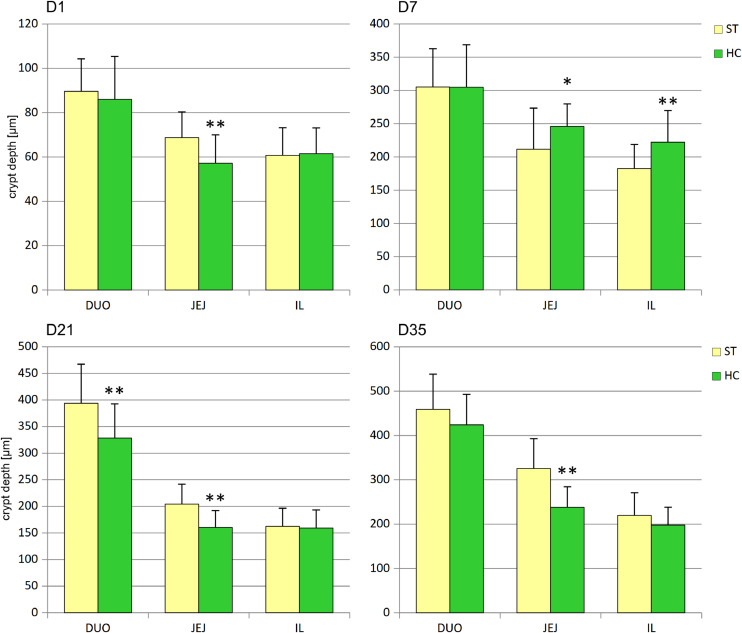
Effect of early feeding on average crypt depths in duodenum (DUO), jejunum (JEJ), and ileum (IL) (mean ± SD, n = 30). ST – standard, HC – HatchCare (early fed) group, D1 – day 1, D7 – day 7, D21 – day 21, and D35 – day 35 after hatching. Significant difference between groups (* *P* < 0.05; ** *P* < 0.01).

Chickens from the HC group began the experiment (D1) with shallower crypts in the jejunum; however, by day 7, crypt depth in the jejunum and ileum had increased significantly (Fig. 2). In contrast, on day 21, crypt depth in the duodenum and jejunum decreased, a pattern that persisted in the jejunum on day 35.

**Fig. 3:**
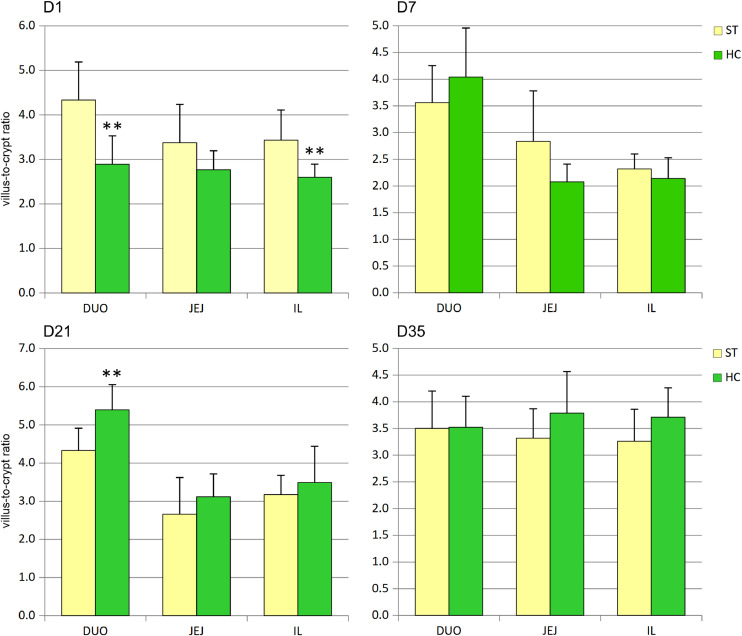
Effect of early feeding on villus-to-crypt ratios in duodenum (DUO), jejunum (JEJ), and ileum (IL) (mean ± SD, n = 30). ST – standard, HC – HatchCare (early fed) group, D1 – day 1, D7 – day 7, D21 – day 21, and D35 – day 35 after hatching. Significant difference between groups (* *P* < 0.05; ** *P* < 0.01).

Early feeding also influenced VCRs (Fig. 3) but did not show a clear trend over the study period. A decrease in VCR of the HC group was observed on day 1 in the duodenum and ileum, while an increase was noted on day 21 in the duodenum. By day 35, VCR values did not differ significantly between HC and ST groups in all segments studied.

**Fig. 4:**
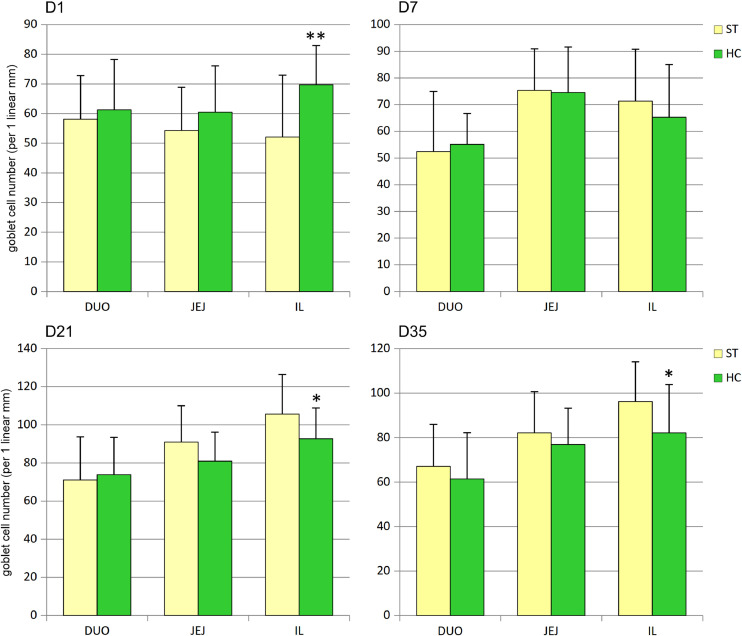
Effect of early feeding on goblet cell numbers (per 1 linear mm) in duodenum (DUO), jejunum (JEJ), and ileum (IL) (mean ± SD, n = 30). ST – standard, HC – HatchCare (early fed) group, D1 – day 1, D7 – day 7, D21 – day 21, and D35 – day 35 after hatching. Significant difference between groups (* *P* < 0.05; ** *P* < 0.01).

The number of goblet cells in the ileum of the HC group was initially (D1) significantly higher but decreased on days 21 and 35 (Fig. 4). In both the duodenum and jejunum, goblet cell numbers did not differ between groups at any time point studied.

## Discussion

Under commercial production conditions, broiler chickens are capable of increasing their BW by up to 60 times their hatching weight by the time they reach slaughter age, with an average daily growth rate of nearly 70 g per day over 48 days (Torrey et al., 2021).

Delayed feeding shortly after hatching reduces final BW and causes several side effects, as outlined in the introduction. To minimize these effects and achieve the full potential of broilers, several early nutritional strategies have been proposed. These include supplying nutrients to the developing embryo before hatch, providing feed within the hatchery, and developing highly digestible prestarter diets (Noy and Uni, 2010).

Previous studies have shown that relative small intestinal weight increases rapidly in broilers during the first week after hatch, then plateaus relative to the chick’s BW (Sklan, 2001). Wang et al. (2020) observed higher absolute and relative small intestinal weights in Lingnan Yellow broilers with immediate access to feed and water compared to groups that fasted for 24 or 48 h. Our study indicated that early feeding improved the length of small intestinal segments on day 1 and increased intestinal weight on day 7, but this effect was limited to individual sections of the small intestine. Moreover, in the long term (D21, D35), these effects did not persist, and even shortening of some intestinal segments was observed. The differences between the results of Wang et al. (2020) and our study are likely due to differences in experimental design. Namely, the beneficial effect of early feeding is clearly visible when fasting birds are directly compared with those receiving feed and water immediately after hatch. However, these differences are less apparent in commercial settings, where birds hatched under standard conditions include individuals that fasted anywhere from 0 to as long as 55 h.

The nutrients stored in the yolk sac contribute to body weight gain in the first days after hatch (van der Wagt et al. 2020). In the first three days after hatching, the contribution of the residual yolk in the total dietary energy and protein intake ranges from 10-11 % (Wijtten, 2011) to 30 % (Murakami et al., 1992). Previous studies found that chickens with immediate access to feed after hatch have lower residual yolk weights at 96 h after hatch than those exposed to the 48-72 h hatching window (Noy et al., 1996; El-Husseiny et al., 2008). It is suggested that higher yolk utilization in early-fed chickens might be related to a higher intestinal activity, possibly resulting from peristaltic movements (van der Wagt et al. 2020). However, other authors obtained opposite results, indicating that yolk utilization or residual yolk weights did not differ between chickens that had immediate or delayed (up to 72 h) access to the feed (Gonzales et al., 2003; Van den Brand et al., 2010). Our study confirmed these latter reports demonstrating that early feeding did not influence the weight and index of the yolk sacs.

The rate of intestinal villi development in chickens exhibits variable dynamics. The greatest increase in villus height occurs during the first week after hatching (Uni et al., 1995, 1998). During this period, the duodenum shows the highest rate of relative growth, followed by the ileum and then the jejunum (Dror et al., 1977). After the first week, villus height continues to increase at least until the typical slaughter age of broilers, i.e., 5 to 6 weeks after hatching (Gabriel et al., 2008; Iji et al., 2001; Miles et al., 2006), with the most pronounced growth observed in the jejunum and ileum (Wijtten et al., 2012). Longer villi have been shown to correlate with increased cell mitosis (Samanya and Yamauchi, 2002). One of the main factors promoting villus height by stimulating enterocyte proliferation, migration, and lifespan is bacterial colonization of the intestine (Wijtten et al., 2012; Willing and Van Kessel, 2007). However, bacteria and other factors causing local or systemic inflammation result in reduced small intestinal villus height (Lee et al., 2010; Peuhkuri et al., 2010). In the current study, villus height increased with age in both groups in the jejunum and ileum until the end of the experiment, while in the duodenum, this parameter decreased starting from day 21. This led to a steady increase in VSA in all gut segments, supporting previous observations by other authors (Gabriel et al., 2008; Iji et al., 2001; Miles et al., 2006).

Comparative analysis between the groups revealed that on day 1 in the duodenum, the height and surface area of intestinal villi were significantly lower in the HC group than in the ST group. However, by day 7, these proportions reversed, with significantly higher values observed in the HC group. It is difficult to clearly explain why villus height and surface area differed between the groups on D1, but these should be regarded as baseline values. Indeed, it is unlikely that villus height changed so markedly within a few hours after hatching as a result of early access to feed. However, the effect of early feeding is clearly visible on D7, indicating that early access to feed stimulates intestinal villus development during the first week of life. These observations are consistent with previous studies by Ao et al. (2012), which showed that early feeding of Cobb broiler chicks increases villus height in the jejunum, but not in the ileum, on day 14 after hatching. Other studies by Wang et al. (2020) and Li et al. (2022), conducted on Lingnan Yellow broilers, revealed that early feeding increases villus height in the jejunum and ileum at various time points during the first week after hatching, as well as in the duodenum on days 21 and 50 after hatching, compared to a 48-h fasting group. Some differences were also observed when compared to the 24-h fasting group. Studies of Shani et al (2024) indicated that direct access to feed led to an increase in villus height, crypt depth in the ileum of 12-day-old broiler chickens. However, the data from all the above-mentioned studies covered different time points, broiler strains, early feeding systems (where access to feed and water was provided outside the hatchery about 2 h after hatching), and distinct experimental designs (fasting birds versus standard conditions) compared to our study.

Another parameter describing the development and function of the small intestine is crypt depth. This is where stem cells proliferate and generate new cells that, after division, either migrate to the bottom of the crypt or to the top of the villus. The latter differentiate into enterocytes (absorptive cells), goblet cells (mucus-producing), and enteroendocrine cells (hormone-producing). It should be noted that, unlike mammals, chicken enterocyte proliferation is not localized solely in the crypt region but also occurs—albeit with lower activity—along the villi (Uni et al., 1998). The lifespan of enterocytes in broilers depends on age, ranging from 2 to 5 days at 1–2 days after hatching to 10 days by 6 months of age (Wijtten et al., 2012). Since enterocyte lifespan increases with age, villus height shows the same tendency. Increased crypt depth indicates high cell turnover allowing intestinal villus renewal (Yason et al., 1987) as well as increased mucosal secretion (Chiou et al., 1996). The present study indicates that, compared to the ST group, early feeding initially increased crypt depth in the jejunum and ileum during the first week of age, followed by a decrease in the jejunum on days 21 and 35. Because new generations of enterocytes developing in the crypts on D7 move quickly to the villus surface, it can be assumed that the increased crypt depth observed in the ileum of the HC group resulted in increased villus height at this age. In contrast, in the jejunum, increased crypt depth on D7 did not affect villus height but significantly increased villus width. Our results do not support previous studies indicating that fasting negatively affects villus height and crypt depth in the small intestine of broilers assessed at market age (42 days after hatching), compared to chicks fed immediately after placement (Gonzales et al., 2003). The absence of a negative effect of starvation on crypt depth at days 21 and 50 after hatching was also demonstrated by Li et al. (2022).

The pattern of villus height to crypt depth ratios differs from that of villus height and crypt depth alone. These ratios were initially lower in the HC group on day 1 in the duodenum and ileum but did not differ between groups at subsequent time points in any small intestinal segment. Only in the duodenum on day 21 was this parameter higher in the HC group than in the ST group, resulting from a shallowing of the crypt depth at this time point. Similar results, although limited only to the ileum and to day 12 after hatching, were obtained by Shani et al. (2024) who found that early feeding does not influence the VCR ratio in broiler chickens. A decrease in VCR usually results from damage or atrophy of the intestinal villi caused by various etiologies, often accompanied by regenerative elongation of the crypts (Erben et al., 2014; Mandile et al., 2022). Conversely, an increase in VCR indicates elevated epithelial cell turnover and/or increased mitosis in the crypts, which is generally beneficial for the bird (Awad et al., 2008; Nguyen et al., 2021).

The mucus secreted by goblet cells is a key component of the intestinal barrier and plays an important role in preventing pathogen invasion in the gastrointestinal tract (Matur et al., 2012). Previous studies of Shani et al (2024) indicated that early access to the feed increases goblet cell density in the ileum of 12-day-old broiler chickens. Our results indicate that early feeding has a limited influence on goblet cell number, noticeable only in the ileum. However, it is difficult to clearly explain why the number of goblet cells in the HC group, which was higher on day 1, decreased in subsequent time points to levels lower than those in the ST group.

Our previous studies indicated that early feeding not only improves broiler production parameters but also enhances their welfare (Gaweł et al., 2022; Madej et al., 2024, 2025). The results of the present study show that early feeding has the most beneficial effects during the first week after hatching. During this period, it stimulates intestinal villus development in the duodenum and ileum, increasing the average VSA in these gut segments. It also increases crypt depth in the jejunum and ileum on day 7. However, these effects did not persist until the end of broiler life cycle.

In conclusion, our findings indicate that early feeding has the most beneficial effects on small intestine development during the first week after hatching. However, these effects did not persist until the end of the rearing period.

## CRediT authorship contribution statement

**Andrzej Gaweł:** Writing – review & editing, Supervision, Resources, Project administration, Methodology, Funding acquisition, Conceptualization. **Kamila Bobrek:** Writing – original draft, Project administration, Investigation, Data curation, Conceptualization. **Jan P. Madej:** Writing – original draft, Visualization, Methodology, Investigation, Data curation, Conceptualization.

## Disclosures

The authors declare the following financial interests/personal relationships which may be considered as potential competing interests: Andrzej Gawel reports financial support was provided by The Agency for Restructuring and Modernisation of Agriculture (Poland). Kamila Bobrek reports financial support was provided by The Agency for Restructuring and Modernisation of Agriculture (Poland). Jan P. Madej reports financial support was provided by The Agency for Restructuring and Modernisation of Agriculture (Poland). If there are other authors, they declare that they have no known competing financial interests or personal relationships that could have appeared to influence the work reported in this paper.
